# Commencement in the University of Buffalo

**Published:** 1862-03

**Authors:** 


### EDITORIAL DEPARTMENT.

### COMMENCEMENT IN THE UNIVERSITY OF BUFFALO.

The Annual Commencement of the Medical Department of the University of Buffalo took place on the evening of February 25, 1862. The exercises were held in American Hall, which was filled to its utmost capacity with a highly respectable and intelligent audience.

The degree of *Doctor in Medicine* was conferred on the following gentlemen by HON. MILLARD FILLMORE, Chancellor of the University, who accompanied the ceremony by a brief and appropriate address to the citizens of Buffalo and the graduating class, viz:

John Winthrop Stille, Otsego, Otsego co., N. Y.
Thomas Boyle Minchen, Adrian, Lenawee co., Mich.
William Robinson, Buffalo, N. Y.
Samuel William Wetmore, Kingsville, Ashtabula co., O.
Delos White Harrington, Akron, Erie co., N. Y.
John Russell Stewart, North Cohocton, Steuben co., N. Y.
P. Henry Clark, Ashland, Ashland co., O.
Charles Gray Robertson, Waterford, Oakland co., Mich.
Elias Sharp Chapel, New Lyme, Ashtabula co., O.
Abel Goodrich Rathbone, New Lyme, Ashtabula co., O.
Horace Bradley Northrop, Arcade, Wyoming co., N. Y.
Solomon V. Frame 2d, Depauville, Jefferson co., N. Y.
John Peter Blawis, Fort Miller, Washington co., N. Y.
Eugene Adelbert Chapman, Henderson, Jefferson co., N.Y.
John William Goodson, Bellevue, Huron co., O.
Theodore Woodard Barton, Waterford, Erie co., Pa.
Oscar Eugene Wainwright, Akron, Erie co., N.Y.
John Cole, Lancaster, Erie co., N. Y.
James Sedequest Smith, Burford, Brant co., C. W.
Edward Little, Newberry, Middlesex co., C.W.
James William Casey, Rochester, N. Y.
Horace Tupper, Buffalo, N. Y.
John Jenkins, Shelby, Orleans co., N. Y.
John Charles Wall, Oshawa, Ontario co., C. W.
Andrew Jackson Haughton, Cambria, Niagara co., N. Y.
James Sprague Wilkin, Shawnee, Niagara co., N. Y.
Charles Weaver Carrier, Newfield, Tompkins co., N. Y.
Charles Washington Colyer, Buffalo, N. Y.

The Faculty deem the following Thesis worthy of honorable mention, viz: a Thesis on "Hygiene," by Andrew Jackson Houghton, of Cambria, Niagara Co., N. Y., and a Thesis on "Sulphate of Quiniæ," by Samuel William Wetmore, of Kingsville, Ashtabula Co., Ohio.

The charge to the graduates by PROF. JAMES P. WHITE was a very able, appropriate and practical address, which we should be happy to publish in full. After an eloquent introduction, of compliment and congratulation, they were exhorted to imprint indelibly upon their minds the conviction that success in life will depend mainly upon themselves; to trust nothing to fortune or fancied position; to labor diligently, to make themselves competent for the performance of every professional duty; to rely chiefly upon their own unaided efforts with inflexible purpose, when they would reach, if not the summit of their ambition, at least an enviable position in life. It was urged that exclusive attention to professional pursuits and worldly objects was wholly unnecessary; an expanded benevolence being in no way incompatible with the true interests of a physician, since it turns away the thoughts for a moment from the schemes of profit or ambition, and more than repays the loss, by its cheering effect upon the heart, and its ennobling influence upon the character.

Motives for the continued prosecution of the study of medicine were presented, and a practitioner who ceases to be a student, represented as no longer worthy the confidence of the public. Whatever may be the attainment, the life of a physician is only truly useful and honorable, when it is unremittingly employed in study. The extensive improvements in the different departments of medical science which are constantly taking place, render it utterly impossible for any practitioner to pursue his avocation with satisfaction, without constant, scrutinizing investigation.

The blissful ignorance of the mere pretender was forcibly contrasted with the care and anxiety which rests upon the mind of the intelligent physician who is painfully alive to the safety and welfare of his patient.

Practical men, about which so much is said at the present time, were shown to be only close students and careful observers, well versed in science. Native vigor of mind might enable one to avoid great mistakes, or possibly obtain a degree of respectability in the profession, but these form the rarest exceptions, and we may well ask, how much more could have been accomplished, with liberal culture, in a mind thus gifted?

The broad field for investigation in Medical Science was happily and truthfully presented, and the enthusiasm often manifested in the investigation and search for truth was most graphically portrayed; the votaries of medical ambition being as often seen, to brave unheeding all the dangers of contagion and infection, and in search of truth becoming in many instances utterly unmindful of their own comforts and necessities. The Surgeons of Bull Run were referred to as instances, who submitted to be taken prisoners rather than leave the wounded under their charge to suffer from neglect, and the thrilling and heroic reply cited of one of their number, who, when asked if he would surrender, with a pistol at his ear to enforce obedience, coolly said, "Yes, when I have tied this artery which is bleeding this poor fellow to death,"

The present graduating class were presented as part of the fruit of the first literary tree of the kind, ever planted in Buffalo, by the generosity and liberality of her citizens; and regarded as conclusive evidence that their beneficence had been judiciously bestowed. It was hoped the prosperous condition of the medical department of the University of Buffalo would arouse a spirit of liberality in the citizens sufficient to organize and establish also on a permanent basis the Academic department of this University. A glowing tribute was here given to the *Alumni* of this Institution, occupying as they do the most useful and responsible positions in life. Their unexceptionable success in obtaining appointments in the Army and Navy; the high places they occupy throughout the country in all the departments of professional life were referred to, as the just pride of their *Alma Mater.*

The class were cautioned to avoid quackery themselves, since quackery is not wholly excluded from the ranks even of the profession or entirely confined to the unlearned; "there are those who know the right, but still the wrong pursue." The nature of medical delusions and the mental organization of those who most greedily imbibe medical error, was truthfully represented. Those who run after nostrums and the various isms, would in other counties, impelled by the same blind faith and ignorance, make their Meccan pilgrimages, to kiss and bow down, to consecrated relics. Public confidence in scientific acquirement, is steadily onward, and advances in exact proportion as the mass of community itself becomes enlightened. To this rule but one exception was made, that of the clergy, who had done more harm than any other class, by giving certificates of cures by quack remedies. This was kindly explained and apologized for, and a most celebrated English Divine quoted: "I have oft times, not without wonder and indignation observed the strange confidence in empyrics in physic, that dare venture on the practice of that noble art which they do not at all understand, considering how for a little paltry gain, they shrewdly hazard, or rather certainly destroy the health and lives of men, and I have judged them worthy of as capital and ignominious punishment, as those who kill men on the highway." The advice and counsel given upon the subject of professional intercourse, was exceedingly well timed and judicious.

This brief review, of some few of the many points in the address, is more unsatisfactory to us, than it can possibly be to any of our readers. Nothing can give a truthful outline short of a full report. It was listened to with marked attention, and many sentiments were greeted by most hearty applause.

After the Commencement exercises, a most sumptuous supper was provided for the Graduates, Curators, Faculty and invited guests, by Prof T. F. Rochester, at the American Hotel. This occasion was one of particular interest; speeches, toasts, &c., together with the last greetings of classmates and teachers, forming in all, a scene, which can be fully appreciated only by those who remember such periods, in their own histories.

